# *Erratum*: Vol. 62, No. RR-1

**DOI:** 10.15585/mmwr.mm695152a7

**Published:** 2021-01-01

In the *MMWR* Recommendations and Reports “Methodology of the Youth Risk Behavior Surveillance System — 2013,” the Republic of the Marshall Islands and the Republic of Palau were erroneously referred to as U.S. territories. Throughout the report, all references to “territories” should have read “territories **and freely associated states**,” and all references to “territorial” should have read “territorial **and freely associated state**.”

